# Physiological and Microscopic Characterization of Cyclic-di-GMP-Mediated Autoaggregation in *Erwinia amylovora*

**DOI:** 10.3389/fmicb.2019.00468

**Published:** 2019-03-12

**Authors:** Roshni R. Kharadi, George W. Sundin

**Affiliations:** Department of Plant, Soil and Microbial Sciences, Michigan State University, East Lansing, MI, United States

**Keywords:** cyclic-di-GMP, *Erwinia amylovora*, aggregation, biofilm, cell division, cell separation, shear stress, laminar flow

## Abstract

The second messenger cyclic-di-GMP (c-di-GMP) is a critical regulator of biofilm formation in the plant pathogen *Erwinia amylovora*. Phosphodiesterase (PDE) enzymes are responsible for the degradation of intracellular c-di-GMP. Previously, we found that the deletion of one or more of the three PDE enzyme encoding genes (*pdeA*, *pdeB*, and *pdeC*) in *E. amylovora* Ea1189 led to an increase in biofilm formation. However, in mutants Ea1189Δ*pdeAC* and Ea1189Δ*pdeABC*, biofilm formation was reduced compared to the other single and double deletion mutants. Here, we attribute this to an autoaggregation phenotype observed in these two mutants. Examination of Ea1189Δ*pdeABC* cellular aggregates using scanning electron microscopy indicated that a subset of cells were impaired in cell separation post cell division. Concomitant with this phenotype, Ea1189Δ*pdeABC* also exhibited increased transcription of the cell-division inhibitor gene *sulA* and reduced transcription of *ftsZ*. Ea1189Δ*pdeABC* showed a significant reduction in biofilm formation, and biofilms formed by Ea1189Δ*pdeABC* exhibited a distinctive morphology of sparsely scattered aggregates rather than an evenly distributed biofilm as observed in WT Ea1189. Our results suggest that highly elevated levels of c-di-GMP lead to increased cell–cell interactions that contribute to autoaggregation and impair cell-surface interaction, negatively affecting biofilm formation.

## Introduction

*Erwinia amylovora* is the causal agent of the globally destructive fire blight disease that affects apple and pear trees and other members of the Rosaceae family (Smits et al., 2017). *E. amylovora* can enter the host through microscopic wounds on shoot tips, following which it can systemically infect the xylem vasculature of leaves. Within the xylem vessels, *E. amylovora* forms extensive biofilms that restrict water transport, which can eventually lead to shoot death, characterized outwardly in the plant as shoot blight symptoms (Norelli et al., 2003; Koczan et al., 2009). Amylovoran, levan, and cellulose are three major exopolysaccharides (EPSs) in *E. amylovora* that contribute to the structure and resilience of the biofilms formed within the xylem (Koczan et al., 2009; Castiblanco and Sundin, 2018).

The ubiquitous bacterial second messenger cyclic-di-GMP (c-di-GMP) is a critical regulator of biofilm formation in *E. amylovora* (Edmunds et al., 2013). Levels of c-di-GMP within cells are controlled by the activity of diguanylate cyclase (DGC) enzymes with GGDEF domains, that function in the synthesis of c-di-GMP, and phosphodiesterase (PDE) enzymes with EAL or HD-GYP domains, that degrade c-di-GMP into 5′-phosphoguanylyl-(3′ → 5′) – guanosine (pGpG) or GMP, respectively (Jenal et al., 2017). Quantitative increases in the intracellular levels of c-di-GMP in *E. amylovora* lead to an increase in the production of amylovoran and cellulose, which together contribute to an increase in biofilm formation (Edmunds et al., 2013; Castiblanco and Sundin, 2018; Kharadi et al., 2019).

Bacterial biofilm formation is a dynamic process that involves surface sensing, attachment, EPS synthesis, and consequently the formation of a mature biofilm over the primary attached layer of cells (Wilson et al., 2017). The qualitative and quantitative determination of biofilms *in vitro* is heavily dependent on how the assay conditions impact each of the steps involved in this process. *In planta*, *E. amylovora* is subjected to shear stress due to transpiration flow in the xylem (Lang, 1990; Koczan et al., 2011). Shear stress has been directly implicated in triggering attachment and subsequent biofilm formation mediated by c-di-GMP in *Pseudomonas aeruginosa* and *Xylella fastidiosa* (De La Fuente et al., 2008; Rodesney et al., 2017). In addition, confined flow can alter flagellar rotation, type IV pili mediated attachment, and EPS production (Conrad and Poling-Skutvik, 2018).

In addition to biofilm formation, elevated levels of c-di-GMP have also been implicated in triggering autoaggregation in several bacterial pathosystems. For example, in *Yersinia pestis*, autoaggregation is regulated by c-di-GMP independently of extracellular matrix production and biofilm formation and is dependent upon the enzymatic activity of the DGC enzyme encoded by gene *hmsT* (Sun et al., 2011). In *Burkholderia pseudomallei*, the deletion of *cdpA*, which encodes a PDE enzyme, results in an increase in c-di-GMP levels that leads to increases in EPS production, biofilm formation, and cell–cell aggregation that subsequently leads to a significant reduction in the ability of *B. pseudomallei* to invade human lung epithelial cells and impact the overall cytotoxicity in human macrophage cells (Lee et al., 2010). In *P. aeruginosa*, a putative DGC enzyme-encoding gene *siaD* was shown to trigger autoaggregation in response to SDS (detergent) induced stress, thus increasing rates of fitness and survival compared to their suspended/non-aggregated counterparts (Klebensberger et al., 2009). Thus, evidence suggests that bacterial autoaggregation can often be triggered by increased levels of c-di-GMP and can consequently impact virulence as well as survival and fitness, with potential long-term evolutionary effects. Also, in certain cases, biofilm formation and autoaggregation are largely independent processes that mainly overlap in the involvement of c-di-GMP in their control.

Cyclic-di-GMP also plays a direct and critical role in cell division and cell cycle progression in some bacteria. For example, *Caulobacter crescentus* undergoes an asymmetrical cell division process, wherein a detached swarmer cell emerges from an attached stalked cell. The spatial distribution of c-di-GMP in both the stalked and swarmer cells varies greatly as the cell cycle progresses (Christen et al., 2010), and the lack of c-di-GMP leads to the formation of elongated cells with mis-located division septa (Abel et al., 2013). In *Streptomyces venezuelae*, c-di-GMP controls the dimerization of the critical growth, sporulation, and cell cycle regulator BldD; in addition, a significant increase or decrease in c-di-GMP levels leads to severe changes in the morphology and the sporulation ability of cells (Tschowri et al., 2014).

In our previous study, we investigated the role of the three PDE enzyme-encoding genes *pdeA*, *pdeB*, and *pdeC* in c-di-GMP degradation and biofilm formation in *E. amylovora* using a combination of single, double and triple *pde* gene knockout mutants (Kharadi et al., 2019). Due to an increase in the intracellular levels of c-di-GMP in all of the *pde* deletion mutants, biofilm formation was significantly elevated in these mutants compared to WT Ea1189. However, in Ea1189Δ*pdeAC* and Ea1189Δ*pdeABC*, although c-di-GMP levels were the highest among all the mutants, a significant reduction in biofilm formation was observed compared to the other single and double mutants (Kharadi et al., 2019). Here, we show that Ea1189Δ*pdeAC* and Ea1189Δ*pdeABC* display an autoaggregation phenotype when grown in liquid medium. Based on existing evidence that implicates c-di-GMP in autoaggregation, our primary hypothesis is that the increased levels of c-di-GMP resulting from the loss of PDE activity in mutants Ea1189Δ*pdeAC* and Ea1189Δ*pdeABC* caused the autoaggregation phenotype in these strains. We also hypothesized that since autoaggregation mainly occurred as a result of cell-cell interactions, the cells that are sequestered within an aggregate would be impaired in their ability to form biofilms.

To examine the validity of our hypotheses, we characterized the autoaggregation phenotype physiologically and microscopically in *E. amylovora*. We examined the structure of Ea1189Δ*pdeABC* aggregates and the morphologies of individual cells within them. We examined the ability of the *pde* gene mutants to form biofilms under relatively static conditions or under confined continuous flow. Based on our initial findings, we also examined the effect of elevated intracellular c-di-GMP levels on cell division and cell-separation post division.

## Materials and Methods

### Bacterial Strains, Plasmids, and Growth Conditions

The relevant characteristics of all bacterial strains, plasmids utilized in this study are listed in Table 1. *E. amylovora* and *Escherichia coli* strains were grown in Luria-Bertani (LB) liquid and agar medium at 28 and 37°C, respectively. Media were amended with the appropriate antibiotics at the following concentrations: ampicillin (Ap; 100 μg/ml), chloramphenicol (Cm; 10 μg/ml), gentamicin (Gm; 10 μg/ml), kanamycin (Km; 100 μg/ml), or tetracycline (Tc; 10 μg/ml).

**Table 1:** Bacterial strains and plasmids used in this study and their relevant characteristics.

Bacterial strain or plasmid	Relevant characteristic(s)	Source or reference
**Strains**		
*E. amylovora*		
Ea1189	Wild type	Edmunds et al., 2013
Ea1189Δ*pdeA*	Deletion of EAM_RS10800 (*pdeA*)	Kharadi et al., 2019
Ea1189Δ*pdeB*	Deletion of EAM_RS16275 (*pdeB*)	Kharadi et al., 2019
Ea1189Δ*pdeC*	Deletion of EAM_RS16620 (*pdeC*)	Kharadi et al., 2019
Ea1189Δ*pdeAC*	*pdeA* and *pdeC* gene deletion mutants	Kharadi et al., 2019
Ea1189Δ*pdeAB*	*pdeA* and *pdeB* gene deletion mutants	Kharadi et al., 2019
Ea1189Δ*pdeBC*	*pdeB* and *pdeC* gene deletion mutants	Kharadi et al., 2019
Ea1189Δ*pdeABC*	*pdeA*, *pdeB*, and *pdeC* gene deletion mutants	Kharadi et al., 2019
Ea1189Δ*pdeABC* Δ*ams*	*pdeA*, *pdeB*, *pdeC* gene deletion mutants combined with *ams* operon deletion mutant	This study
Ea1189Δ*pdeABC* Δ*bcsA*	*pdeA*, *pdeB*, *pdeC*, and *bcsA* gene deletion mutants	This study
Ea1189Δ*pdeABC* Δ*ams* Δ*bcsA*	*pdeA*, *pdeB*, *pdeC*, and *bcsA* gene deletion mutants combined with *ams* operon deletion mutant	This study
**Plasmids**		
pKD3	Cm^r^ cassette flanking FRT sites; Cm^r^	Datsenko and Wanner, 2000
pKD46	L-Arabinose-inducible lambda red recombinase; Ap^r^	Datsenko and Wanner, 2000
pTL18	IPTG-inducible FLPase; Tet^r^	Long et al., 2009
pBBR1MCS-5	Broad-host-range cloning vector; R6K ori; Gm^r^	Kovach et al., 1995
pACYCDuet-1	Expression vector containing two MCS: P15A ori; Cm^r^	Novagen; Darmstadt, Germany
pRRK01	*pdeA* with native promoter in pBBR1MCS-5; Gm^r^	Kharadi et al., 2019
pRRK02	*pdeB* with native promoter in pBBR1MCS-5; Gm^r^	Kharadi et al., 2019
pRRK03	*pdeC* with native promoter in pBBR1MCS-5; Gm^r^	Kharadi et al., 2019
pRRK04	*pdeA* and *pdeB* with their respective native promoters in pACYCDuet-1; Cm^r^	Kharadi et al., 2019
pRRK05	*pdeB* and *pdeC* with their respective native promoters in pACYCDuet-1; Cm^r^	Kharadi et al., 2019
pRRK06	*pdeA* and *pdeC* with their respective native promoters in pACYCDuet-1; Cm^r^	Kharadi et al., 2019
pMP2444	pBBR1MCS-5 expression *gfp* under *lac* promoter, Gm^r^	Stuurman et al., 2000

### Genetic Manipulations and Analyses

The reference genome sequence of *E. amylovora* ATCC 49946 (Sebaihia et al., 2010) was obtained from GenBank (accession no. FN666575), and Artemis (Java) was used to browse the annotated *E. amylovora* genome. Standard protocols were used for DNA manipulations (Sambrook and Russell, 2001), and chromosomal deletions in *E. amylovora* were constructed using the lambda Red recombinase protocol (Datsenko and Wanner, 2000; Zhao et al., 2009). Primers used for the chromosomal deletions of *bcsA* gene and the *ams* operon were referenced in Zhao et al. (2009) and Castiblanco and Sundin (2018), respectively.

### Determination of Aggregation Factor

Bacterial strains were grown for 18 h in liquid culture with shaking; the culture tubes were then maintained statically for 1 h, and 200 μL of growing medium was removed from the culture. Following this, the cultures were vortexed for 30 s, and 200 μL of growing medium was removed from the culture. The aggregation factor was calculated by determining the OD_600_ turbidimetric ratio of the medium removed post-homogenization to that removed pre-homogenization. This assay was conducted three times, with three technical replicates for each biological replicate.

### Analysis of Cell Growth Pattern and *in vitro* Biofilm Formation Using Scanning Electron Microscopy (SEM)

To visualize cell growth patterns in a liquid medium, samples were drawn directly from cultures grown for 18 h, and fixed using 2.5% paraformaldehyde-2.5% glutaraldehyde. To evaluate biofilm formation *in vitro*, bacterial strains were grown overnight and equilibrated to an OD_600_ of 0.5. A sample of 200 μL of the normalized cultures was added to 2 ml of 0.5X LB in individual wells in a 24-well plate containing a 300-mesh gold grid (Electron Microscopy Sciences, Hatfield, PA, United States) and covered with a breathable covering. After incubation under gentle rocking movement at 24°C for 72 h, the grids were fixed using 2.5% paraformaldehyde-2.5% glutaraldehyde. Following fixation, all samples were dehydrated using ethanol at successively increasing concentrations, critical point dried, and osmium coated as previously described for *E. amylovora* (Castiblanco and Sundin, 2018). The samples were imaged in a JEOL JSM-7500F (cold field emission electron emitter) scanning electron microscope (Japan Electron Optics Laboratory Ltd., Tokyo, Japan).

### RNA Isolation and qRT-PCR

Samples were drawn from bacterial strains grown for 18 h for RNA extraction using the Direct-zol RNA Miniprep kit method (Zymo Research, Irvine, CA, United States). C-DNA was synthesized using a TaqMan reverse transcription (RT) kit (Applied Biosystems, Foster City, CA, United States). Quantitative PCR reactions were performed using SYBR green PCR master mix (Applied Biosystems, Foster City, CA, United States); *recA* was used as an endogenous control in gene-expression analyses. These experiments were conducted three times, with three technical replicates in each biological replicate.

### Evaluating Biofilm Formation in Flow Cells Using Confocal Laser Scanning Microscopy

*Erwinia amylovora* strains expressing *gfp* from pMP2444 (Stuurman et al., 2000) were grown for 18 h and equilibrated to an OD_600_ of 0.5. Flow channels in a μ-Slide VI 0.5 glass bottom slide (Ibidi, Martinsried, Germany) were pre-conditioned with LB for 24 h prior to conducting the assay. Normalized cultures were inoculated into individual flow channels and incubated at 24°C for 1 h, following which all inoculum from the channels was flushed out with 0.5X LB. This was followed by either a static incubation or incubation under flow (with 0.5X LB) generated using a peristaltic pump (Ismatec REGLO Digital 4-CH pump (Cole-Parmer; Vernon Hills, IL, United States) for 5 h. Biofilms developed in the flow channels were visualized using a Zeiss 510 Meta ConfoCor3 LSM confocal laser scanning microscope (Carl Zeiss Microimaging, Jena, Germany). Imaging was performed by acquiring Z-stacks of fluorescent bacterial cells in the individual flow channels. ImageJ software (Schneider et al., 2012) was used to obtain three-dimensional representations of the biofilm distributions.

## Results

### Elevated Levels of c-di-GMP Confer an Autoaggregation Phenotype

**FIGURE 1:**
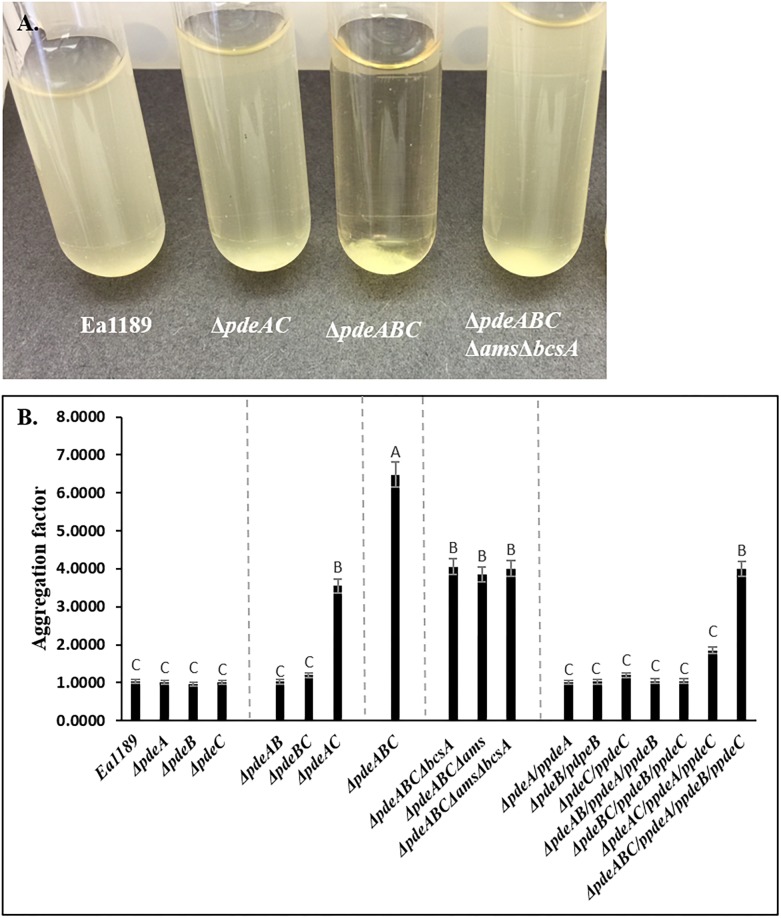
**(A)** *Erwinia amylovora* WT Ea1189, *pde* mutants and complemented mutants Ea1189 grown in LB medium for 18 h with shaking. Image was taken after 1 h of static incubation. **(B)** Calculated aggregation factor for WT *E. amylovora* Ea1189, *pde* mutants, and complemented strains. Data represent three biological replicates, and error bars represent standard errors of the means. Different letters above the bars indicate statistically significant differences [*P* < 0.05 by Tukey’s honestly significant difference (HSD) test].

Ea1189Δ*pdeABC* exhibited a strong autoaggregation phenotype when grown in liquid LB medium; Ea1189Δ*pdeAC* auto-aggregated to a lesser degree than Ea1189Δ*pdeABC*, yet showed distinct signs of autoaggregation compared to WT Ea1189 (Figure 1A,B). In contrast, the WT *E. amylovora* Ea1189, Ea1189Δ*pdeA*, Ea1189Δ*pdeB*, and Ea1189Δ*pdeC* single gene deletion mutants, and Ea1189Δ*pdeAB* and Ea1189Δ*pdeBC* double mutants, did not exhibit autoaggregation when grown in liquid LB medium (Figure 1A,B). Ea1189 Complementation of Ea1189Δ*pdeABC* with all three *pde* genes along with their native promoters (pRRK02 and pRRK06) was able to significantly reduce but not completely eliminate the level of autoaggregation (Figure 1B and Table 1). In order to evaluate if the over-production of amylovoran and/or cellulose under the influence of c-di-GMP was causing autoaggregation, we deleted the *ams* operon and the *bcsA* gene both separately and together in EA1189Δ*pdeABC*. The multiple mutant strains EA1189Δ*pdeABC* Δ*ams*, EA1189Δ*pdeABC* Δ*bcsA*, and EA1189Δ*pdeABC* Δ*bcsA* Δ*ams* all exhibited a marked reduction in autoaggregation (Figure 1A,B).

### Some Cells Within an Aggregate Are Impaired in Cell Separation Post Cell Division

**FIGURE 2:**
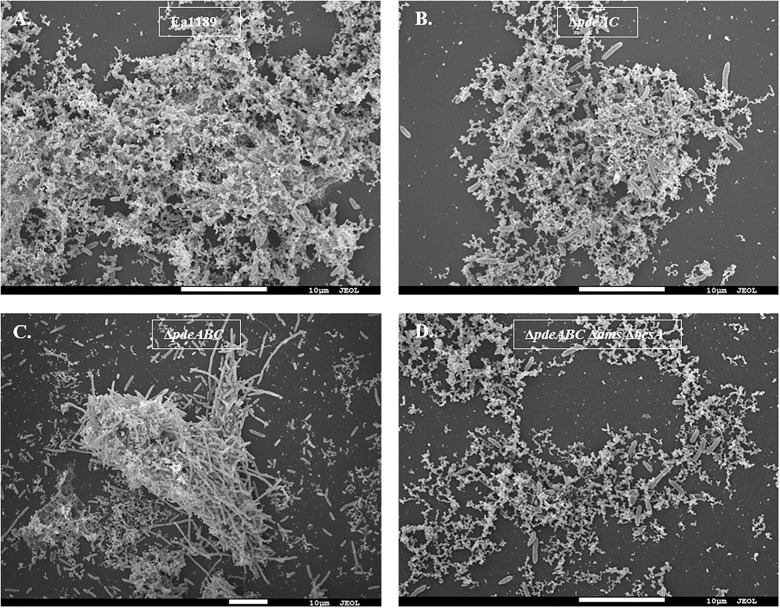
Scanning electron microscopy images of *E. amylovora* strains grown in LB for 18 h. **(A)** WT Ea1189 shows cells embedded in a diffused EPS organization. **(B)** Ea1189Δ*pdeAC* cells show a relatively diffused arrangement. **(C)** Ea1189Δ*pdeABC* cells are highly organized in an aggregate with most EPS bound within it. Elongated cells are interspersed in and around the aggregate. **(D)** Ea1189Δ*pdeABC* Δ*ams* Δ*bcsA* cells show a diffused arrangement with a reduced prevalence of EPS around the cells.

Our microscopic evaluations of strains grown in liquid LB medium revealed a marked distinction between aggregating and non-aggregating strains. WT Ea1189 cells exhibited a diffused arrangement of cells within an extracellular matrix (Figure 2A). In contrast, Ea1189Δ*pdeABC* cells showed a more structured arrangement of cells, with the extracellular matrix being embedded within the aggregate (Figure 2A,C). Ea1189Δ*pdeAC* and Ea1189Δ*pdeABC* Δ*bcsA* Δ*ams* cells showed a relatively unstructured arrangement compared to Ea1189Δ*pdeABC*, however, the cells were more distinctly arranged compared to WT Ea1189 (Figure 2B,D).

**FIGURE 3:**
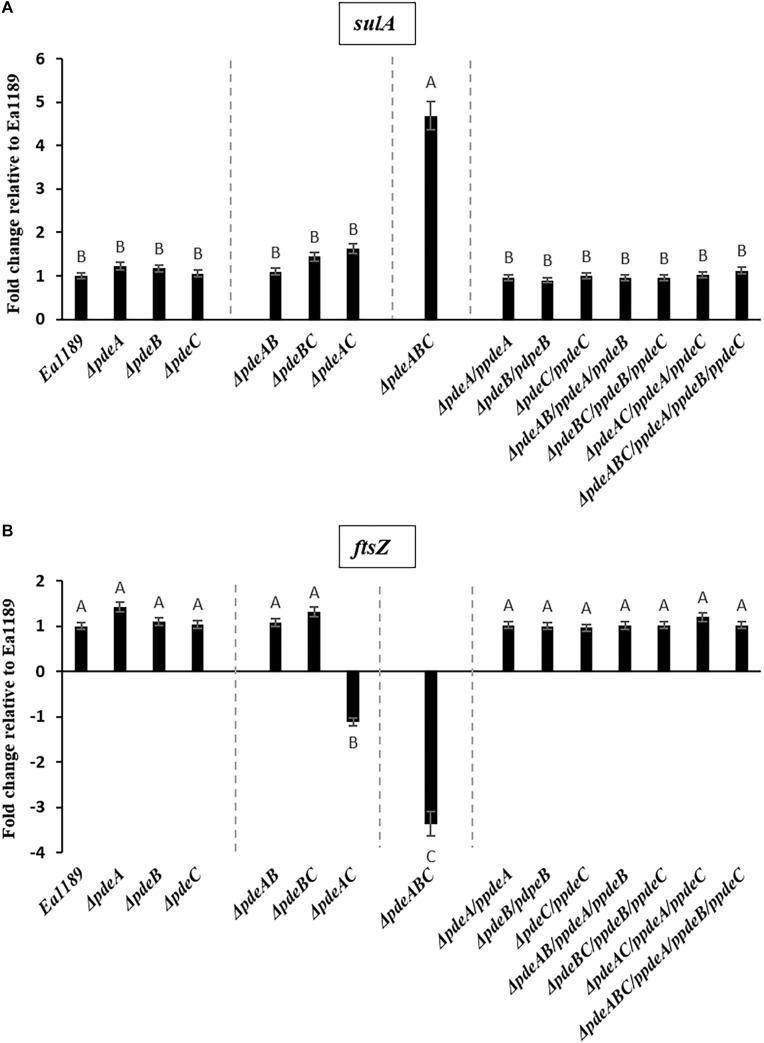
**(A)** *sulA* expression in *E. amylovora* WT Ea1189, *pde* mutants, and complemented mutants grown in LB medium for 18 h. Gene expression for the mutants and complemented strains has been normalized to relative expression levels in Ea1189. Data represents three biological replicates. Different letters above the bars indicate statistically significant differences [*P* < 0.05 by Tukey’s honestly significant difference (HSD) test)]. **(B)** *ftsZ* expression in *E. amylovora* WT Ea1189, *pde* mutants, and complemented mutants grown in LB medium for 18 h. Gene expression for the mutants and complemented strains has been normalized to relative expression levels in Ea1189. Data represents three biological replicates. Different letters above the bars indicate statistically significant differences [*P* < 0.05 by Tukey’s honestly significant difference (HSD) test].

In addition, a subset of the cells within and adjacent to an Ea1189Δ*pdeABC* aggregate were unusually elongated, with some cells several times longer their normal size (Figure 2C). These cells characteristically exhibited the presence of several interspersed division septa throughout the length of the cell (Figure 2C). The localization and the assembly of FtsZ into its characteristic ring-like structure in the cytoskeleton is a key marker of cell division (Bi and Lutkenhaus, 1991). FtsZ is under the control of cell division inhibitor SulA, a component of the bacterial SOS response (Bi and Lutkenhaus, 1993). Using q-RT-PCR, we examined the transcript levels of *sulA* and *ftsZ* in the *pde* mutants grown in LB. We wanted to evaluate if the presence of a sub-population of cells that were unable to separate after dividing within a Ea1189Δ*pdeABC* aggregate, could yield a quantitative difference in the transcript levels of *sulA* and *ftsZ*. Examined in a collective population of cells, both part of and not part of an aggregate, Ea1189Δ*pdeABC* also showed a significantly higher transcription of *sulA* as well as significantly decreased transcription of *ftsZ*, compared to WT Ea1189 and all single and double *pde* mutants (Figure 3A,B). Complementation of all mutants restored *sulA* and *ftsZ* expression to WT Ea1189 levels (Figure 3A,B).

### Autoaggregation Negatively Affects Biofilm Formation Under Static Conditions

**FIGURE 4:**
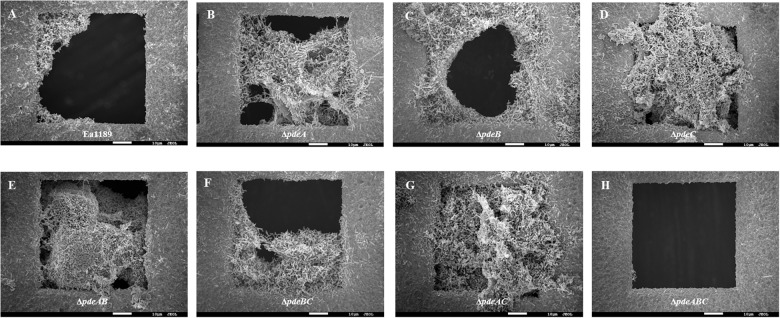
Scanning electron microscopy images showing biofilm formation on gold grids incubated with *E. amylovora* strains for 72 h. **(A)** Ea1189, **(B)** Ea1189Δ*pdeA*, **(C)** Ea1189Δ*pdeB*, **(D)** Ea1189Δ*pdeC*, **(E)** Ea1189Δ*pdeAB*, **(F)** Ea1189Δ*pdeBC*, **(G)** Ea1189Δ*pdeAC*, **(H)** Ea1189Δ*pdeABC*.

Biofilm formation under static conditions on gold-plated grids, was evaluated using SEM, and was shown to be increased in all single and double *pde* mutants compared to WT Ea1189 (Figure 4A–H). In contrast, Ea1189Δ*pdeABC* exhibited significantly reduced biofilm formation on the grids, with only a few cells being able to attach to the grid surface, but unable to develop mature biofilms (Figure 4H). Qualitatively, the biofilms formed by the single and double *pde* deletion mutants were increased in both attachment to the inner edges of the grid subsections as well as in the ability to form robust, mature biofilms, as compared to WT Ea1189 (Figure 4A–G).

### Biofilm Formation Under Shear Stress Is Negatively Impacted by Autoaggregation

**FIGURE 5:**
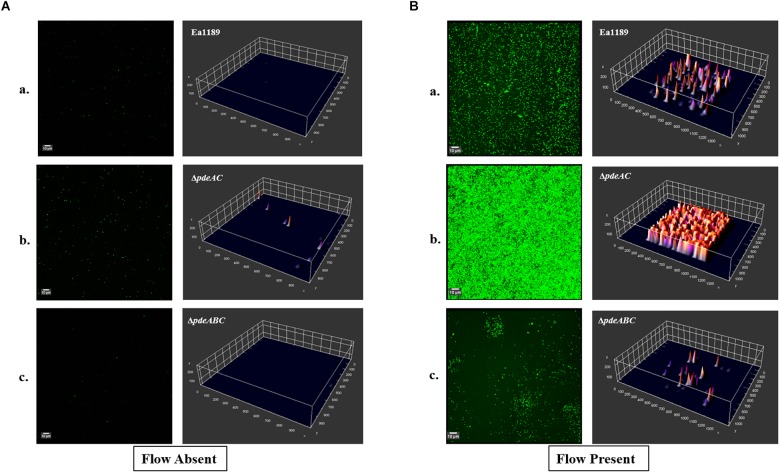
Confocal laser scanning microscopy images showing *E. amylovora* strains expressing *gfp*. Biofilm formation within the flow channels was assessed for **(Aa)** WT Ea1189, **(b)** Ea1189Δ*pdeAC*, and **(c)** Ea1189Δ*pdeABC*, under static conditions with an initial incubation with inoculum for 1 h, followed by a 5 h incubation after the inoculum was flushed out of the flow channel. **(Ba)** WT Ea1189, **(b)** Ea1189Δ*pdeAC*, **(c)** Ea1189Δ*pdeABC*, under conditions of constant confined flow with an initial incubation with inoculum for 1 h, followed by a 5 h continual flow of 0.5X LB through the flow channels. Three dimensional representations of the attached cells and the multilayer biofilms formed by them are presented adjacent to the CLSM Z-stack images (measurements on all axes are presented in μm).

In a flow cell channel, the base of the channel experiences shear stress due to the flow of liquid across its surface. Using WT Ea1189 and *pde* mutants expressing *gfp* encoded on a heterologous vector, we evaluated the ability of the strains to form biofilms in the presence and absence of flow. We used WT Ea1189, Ea1189Δ*pdeAC*, and Ea1189Δ*pdeABC* in this assay, to represent *E. amylovora* strains with low, moderate, and high levels of intracellular c-di-GMP, respectively (Kharadi et al., 2019). Primarily, we found that all three assayed strains showed an increase in the overall level of attachment and multilayer biofilm formation under flow as compared to conditions of no flow (Figure 5A,B). Under flow, Ea1189Δ*pdeAC* cells formed more uniformly robust, multilayered biofilms, as compared to WT Ea1189 (Figure 5Bb). Ea1189Δ*pdeABC* biofilm showed a distinctive pattern of a few areas displaying multilayered, mature biofilms, and otherwise very sparse attachment (Figure 5Bc).

## Discussion

Our results indicate that c-di-GMP is a major, but not the sole contributor to autoaggregation in *E. amylovora*. We found that progressively increasing intracellular levels of c-di-GMP generated by native *E. amylovora* DGC enzymes caused the cells to shift to a state of autoaggregation, once intracellular levels of c-di-GMP reached a certain threshold, as observed in Ea1189Δ*pdeAC*, and beyond in Ea1189Δ*pdeABC*, which showed more severe autoaggregation Ea1189. Thus, we concluded that autoaggregation in *E. amylovora* is dependent on c-di-GMP generated by *E. amylovora* DGC enzymes, whose localization within the cell might provide targeted regulation of autoaggregation. The link between the localized generation of c-di-GMP within the cell and its functionality is well established in other bacterial systems (Schmidt et al., 2005; Römling et al., 2017). Inversely, we observed that the complete complementation of Ea1189Δ*pdeABC*, which restored c-di-GMP back to WT Ea1189 levels, did not eliminate autoaggregation (Figure 1), thus, suggesting that autoaggregation is dependent on both the quantitative increase in c-di-GMP, but, also the specific signaling function of the Edc (*Erwinia* diguanylate cyclase) and Pde enzymes. The autoaggregation phenotype in *E. amylovora* and the similar phenotype reported in *B. pseudomallei* share key similarities in terms of being mediated by increased c-di-GMP levels due to PDE inactivity and the phenotypic appearance of autoaggregation and its severity (Lee et al., 2010).

Our results also suggest that the EPSs amylovoran and cellulose are the other major contributors to autoaggregation in *E. amylovora*. Amylovoran is the predominant EPS, a pathogenicity factor, as well as the major EPS component of biofilms in *E. amylovora* (Koczan et al., 2009). In *E. amylovora*, c-di-GMP positively regulates amylovoran production by activating high levels of transcription of the *ams* operon (Kharadi et al., 2019). In our previous study, we determined that Ea1189Δ*pdeAC* and Ea1189Δ*pdeABC* exhibited the highest levels of both *amsG* (first gene in the *ams* operon) transcripts as well as overall amylovoran production *in vitro*, as compared to all of the other *pde* mutants and WT Ea1189 (Kharadi et al., 2019). We can thus conclude that the increased levels of c-di-GMP in Ea1189Δ*pdeAC* and Ea1189Δ*pdeABC* cause a subsequent increase in amylovoran production, which enhances autoaggregation (Figure 1A,B, 2). Cellulose regulates the overall structure of *E. amylovora* biofilms and positively contributes to virulence *in planta* (Castiblanco and Sundin, 2018). Cellulose production in *E. amylovora* is regulated through the allosteric activation of BcsA via c-di-GMP binding, resulting in activation of cellulose biosynthesis (Castiblanco and Sundin, 2018). Our results suggest that both amylovoran and cellulose contribute quantitatively to autoaggregation; however, the initial increase in c-di-GMP levels in the cell is still indispensable to initiate and maintain autoaggregation. EPS is a also a critical factor for the maintenance of autoaggregation in *P. aeruginosa*, where autoaggregation results due to detergent stress and is a beneficial survival mechanism (Klebensberger et al., 2009). Also, in *Y. pestis*, c-di-GMP is known to positively regulate EPS production, which is critical for cell aggregate formation (Sun et al., 2011).

Using SEM, we were able to characterize the effect of autoaggregation at the microscopic level on the growth pattern of cells in a liquid medium. We found that Ea1189Δ*pdeABC* cells grew in well-defined clusters, unlike the other non-aggregating strains, whose cells were typically scattered evenly through the medium. Cells of the partially aggregating strain Ea1189Δ*pdeAC*, which had lower levels of c-di-GMP compared to Ea1189Δ*pdeABC*, did not show clear aggregate formation. The EPS generated by non-aggregating and partially aggregating cells was distributed fairly evenly in the medium. However, the EPS generated by Ea1189Δ*pdeABC* cells were used to structurally reinforce aggregates, and very little was found unbound by an aggregate in the medium. The recruitment of EPS to structurally support aggregates explains why amylovoran and cellulose are needed for the severe aggregation phenotype observed in Ea1189Δ*pdeABC*. Without amylovoran and cellulose, Ea1189Δ*pdeABC* Δ*bcsA* Δ*ams* cells could not form well defined cellular aggregates. Thus, c-di-GMP and EPS, especially amylovoran and cellulose, are critical for the structural integrity and durability of an *E. amylovora* aggregate.

We also observed that an increase in c-di-GMP levels enabled some cells in and around an Ea1189Δ*pdeABC* aggregate to become unusually elongated, with lengths up to several times their average size. These elongated cells also displayed several interspersed division septa along the length of the cell. Decreased gene expression of *ftsZ* resulting from activity of SulA helps explain the presence of elongated cells that are unable to separate after division (Bi and Lutkenhaus, 1993). We must also acknowledge that q-RT-PCR quantifies the overall average transcript levels of a target within a large group of cells. Our data represent the average change in transcription for all cells in our sample. Based on real time observations of an aggregate with SEM, we concluded that the negative effect of c-di-GMP on cell separation post cell division is unequally distributed in the population. Mechanistic evidence for c-di-GMP mediated negative regulation of cell division comes from *E. coli*, where c-di-GMP was shown to reduce the activity of Lon protease, which regulates SulA activity (Osbourne et al., 2014).

Biofilm formation under relatively static conditions, examined using gold-plated SEM grids showed that progressively increasing levels of c-di-GMP lead to an increase in overall attachment and mature biofilm formation. However, once c-di-GMP levels reached the threshold that was the demarcation between the presence and absence of autoaggregation, biofilm formation was severely impaired. We attribute this to Ea1189Δ*pdeABC* forming and existing as aggregates in solution. The elevated cell-cell interaction that results in autoaggregation, we conclude, impairs the ability of cells to make contact with and attach to a surface, and subsequently to form and sustain multi-layered biofilms, under relatively static conditions. Our observations are in contrast to the results of other studies that correlate autoaggregation with increased biofilm formation phenotypes in organisms such as *P. aeruginosa* and *Sinorhizobium meliloti* (Sorroche et al., 2012; Kragh et al., 2016). Our findings in *E. amylovora* corroborate the known effect of c-di-GMP on biofilms, however, we were able to establish that the impact of c-di-GMP on biofilm formation is not unilateral, and instead is very sensitive to quantitative changes in intracellular c-di-GMP (Edmunds et al., 2013).

Through a systematic comparison of biofilm formation in the presence and absence of confined flow, we found that attachment and biofilm formation were significantly increased under conditions of continual flow as compared to static conditions. This effect was independent of the c-di-GMP levels within a strain and the tendency to aggregate. However, c-di-GMP significantly enhanced the ability to attach and form robust, multilayered biofilms under the shear stress resulting from confined flow. Evidence shows that in *P. aeruginosa*, type IV pili, under the regulation of c-di-GMP, are involved in surface mechanosensing and attachment (Rodesney et al., 2017). Our evidence suggests that overall, autoaggregation negatively affects the ability of Ea1189Δ*pdeABC* to attach to a surface and form multilayered biofilms, with or without the impact of flow. This effect is amplified under static conditions, wherein a large population of cells are sequestered in an aggregate and are unable to attach to a surface. Under flow, however, the cells that are able to attach to a surface form multi-layered biofilms in discrete pockets on the surface of flow cells. This finding might be indicative of the evolution of *E. amylovora* to form biofilms within the xylem, under the constant shear stress from water movement (Lang, 1990; Koczan et al., 2009).

In summary, we found that there is a natural gradient in the impact of intracellular levels of c-di-GMP on cell-cell interactions in *E. amylovora*, cell growth in a liquid medium, cell division, cell separation, and most importantly, on biofilm formation. Increasing levels of c-di-GMP positively impact attachment and biofilm formation until they reach a certain threshold, as evidenced in Ea1189Δ*pdeAC*. Beyond this, as observed in Ea1189Δ*pdeABC*, any increase in c-di-GMP levels causes increased cell-cell interaction that results in cells growing in the form of aggregates in solution. Amylovoran and cellulose also contribute quantitatively to autoaggregation. In addition, the elevated levels of c-di-GMP negatively impact cell division and cell separation post division, with varying severity across the population. The predominance of aggregates in a relatively static solution can negatively impact how cells come in contact with a surface, attach, and form a multilayered biofilm. In the presence of flow, however, *E. amylovora* can form more robust biofilms, as compared to static conditions, and, this difference is most significant in Ea1189Δ*pdeABC*, which, despite autoaggregation, is able to recover from its impairment in biofilm formation under static conditions, and, can form multilayered biofilms under flow. Autoaggregation of bacterial cells can be mediated by a variety of genes, including genes encoding type IV pili, fimbriae, or flagella, as well as large adhesin proteins and small β-barrel proteins (De La Fuente et al., 2008; Trunk et al., 2018). In order to gain a more complete understanding of autoaggregation in *E. amylovora*, it will be necessary to identify cellular determinants of autoaggregation as well as characterize the effects of c-di-GMP on their function.

## Data Availability

The datasets generated for this study are available on request to the corresponding author.

## Author Contributions

RK and GS conceived the research and planned the experimental outline. RK performed the experiments and wrote the manuscript. GS reviewed and edited the manuscript.

## Conflict of Interest Statement

The authors declare that the research was conducted in the absence of any commercial or financial relationships that could be construed as a potential conflict of interest.
